# Citicoline May Effectively Reduce Hard Exudates in Diabetic Retinopathy

**DOI:** 10.3390/biomedicines13102358

**Published:** 2025-09-26

**Authors:** Martina Tomić, Toma Babić, Tomislav Bulum, Spomenka Ljubić, Tomislav Jukić

**Affiliations:** 1 Department of Diabetic Eye Complications, Vuk Vrhovac University Clinic for Diabetes, Endocrinology and Metabolic Diseases, Merkur University Hospital, 10000 Zagreb, Croatia; 2 Department of Diabetes and Endocrinology, Vuk Vrhovac University Clinic for Diabetes, Endocrinology and Metabolic Diseases, Merkur University Hospital, 10000 Zagreb, Croatia; 3 School of Medicine, University of Zagreb, 10000 Zagreb, Croatia; 4 Department of Ophthalmology, Zagreb University Hospital Center, 10000 Zagreb, Croatia

**Keywords:** nonproliferative diabetic retinopathy, hard exudates, glutamate, citicoline, retinal neuroprotection

## Abstract

**Background/Objectives:** Diabetic retinopathy (DR) develops from the interplay of vascular, inflammatory, and neurodegenerative processes. Citicoline, a natural compound essential for cell membranes, enhances neurotransmitter levels, has a neuroprotective effect, reduces oxidative stress by increasing glutathione, and decreases glutamate toxicity. Studies suggest that a citicoline liposomal formulation (eye drops) may prevent diabetes-induced retinal neurodegeneration. This study aimed to evaluate the impact of citicoline eye drops on the clinical signs of DR in clinical settings. **Methods:** More than 100 patients with nonproliferative DR (NPDR) were selected consecutively from the DR screening program and included in this real-life prospective observational clinical study. Each patient underwent color-fundus photography of two fields (macular field and disk/nasal field) in both eyes using a standard 45° fundus camera. Patients were prescribed citicoline eye drops and followed for a period of one year or longer. **Results:** In 4 patients with NPDR and macular hard exudates, the application of citicoline (Omk1^®^) eye drops three times a day for at least six months to a year resulted in a reduction or complete disappearance of hard exudates. **Conclusions:** Our study, to the best of our knowledge, is the first one that establishes a clinically positive effect of citicoline eye drops on hard exudates in DR. However, to support the potential value of citicoline in the treatment of DR, the conclusions of this study still need to be confirmed by statistical analysis of a larger sample size and prospective studies with longer follow-up periods.

## 1. Introduction

### 1.1. Background

Diabetic retinopathy (DR) is a progressive diabetic eye complication that affects the retina, finally leading to visual impairment and blindness if untreated. It is one of the leading causes of preventable blindness worldwide, making it a critical public health issue [1]. DR affects a significant proportion of individuals with diabetes globally. According to the International Diabetes Federation (IDF), there were 588.7 million individuals living with diabetes in 2024, a figure projected to increase dramatically by 2045 [2]. Approximately one-third of these individuals develop diabetic retinopathy, with a significant percentage progressing to vision-threatening complications such as diabetic macular edema (DME) or proliferative diabetic retinopathy (PDR) [3].

### 1.2. The Pathogenesis of Diabetic Retinopathy

The pathogenesis of DR is complex and multifactorial, involving a combination of vascular, inflammatory, and neurodegenerative processes [4,5,6]. Chronic hyperglycemia is its primary driver. It induces oxidative stress through the overproduction of reactive oxygen species (ROS), which damage retinal endothelial cells and pericytes. The loss of pericytes, crucial for maintaining vascular stability, results in capillary leakage and the formation of microaneurysms. Furthermore, hyperglycemia triggers the activation of inflammatory pathways, including the release of cytokines such as vascular endothelial growth factor (VEGF) and tumor necrosis factor-alpha (TNF-α). VEGF, in particular, plays a pivotal role in promoting angiogenesis and vascular permeability, leading to retinal edema and the formation of fragile new blood vessels in PDR. In addition to these vascular abnormalities, DR involves retinal neurodegeneration [7,8]. Retinal ganglion cells and photoreceptors are damaged by a combination of metabolic stress, glutamate excitotoxicity, and inflammation. This neurodegenerative component often precedes detectable vascular changes and contributes to visual dysfunction [9].

### 1.3. The Role of Glutamate in Diabetic Retinopathy

Glutamate, the primary excitatory neurotransmitter in the retina, plays a significant role in the pathophysiology of DR [10,11]. While essential for normal retinal function, excessive extracellular glutamate levels can contribute to neurodegeneration through a process known as excitotoxicity [12]. Diabetes disrupts glutamate metabolism by impairing the function of Müller cells, which are responsible for the uptake and recycling of glutamate. As a result, glutamate accumulates in the extracellular space, where it overstimulates glutamate receptors such as N-methyl-D-aspartate (NMDA) receptors. Overactivation of NMDA receptors leads to an excessive influx of calcium into neurons, triggering a cascade of intracellular events that ultimately results in cell death. This process, known as excitotoxicity, particularly affects retinal ganglion cells and contributes to the neurodegenerative features of DR [13].

### 1.4. Hard Exudates in Diabetic Retinopathy

Among the clinically prominent hallmarks of DR are hard exudates (HE), the lipid and protein deposits that appear as yellowish lesions on the retina [14]. These deposits are often clustered around retinal microaneurysms or areas of vascular leakage and serve as a clear sign of advanced retinal pathology, particularly when they accumulate in or around the macula, leading to DME, the primary cause of vision loss in individuals with diabetes [15]. The formation of hard exudates in the retina begins with hyperglycemia, which damages the retinal capillaries and disrupts the blood-retinal barrier (BRB), leading to increased vascular permeability. This allows plasma components, particularly lipids from low-density lipoproteins (LDL) and very low-density lipoproteins (VLDL), to leak into the retinal tissue. Under normal conditions, these lipoproteins are cleared by phagocytic cells; however, in diabetes, chronic inflammation impairs this clearance, leading to the accumulation of deposits. Furthermore, chronic inflammation, characterized by elevated pro-inflammatory cytokines such as interleukin-6 (IL-6) and TNF-α, exacerbates vascular leakage and recruits immune cells, thereby contributing to tissue damage. VEGF also plays a significant role. Besides promoting neovascularization, it also increases vascular permeability, thereby worsening the leakage of plasma lipoproteins into the retina. Additionally, the extracellular matrix (ECM) of the retina undergoes significant remodeling in diabetes, with proteins such as fibronectin and laminin accumulating in the ECM, thereby promoting lipoprotein retention, while matrix metalloproteinases (MMPs), enzymes responsible for ECM turnover, also dysregulated in diabetes, further alter the retinal structure and stimulate the formation of deposits [16,17].

This study aimed to evaluate the impact of citicoline eye drops on the clinical signs of DR in clinical settings, as part of everyday retinological practice.

## 2. Patients and Methods

### 2.1. Study Design and Ethics

This real-life prospective observational clinical study was conducted in Vuk Vrhovac University Clinic for Diabetes, Endocrinology and Metabolic Diseases, Merkur University Hospital in Zagreb, Croatia, following the Declaration of Helsinki and approved by the Hospital’s Ethics Committee (protocol number 06/115-19, approval date: 15 June 2019). All study participants were orally informed about the study protocol and signed a written informed consent form.

### 2.2. Patients

More than 100 patients, both genders, and older than 18 years with T1DM and T2DM and NPDR, were selected consecutively from the diabetic retinopathy screening program and included in the study by the first author, M.T., during her routine clinical work. At the inclusion visit, after signing the informed consent, the first author obtained a medical history regarding the duration of diabetes and other eye conditions and diseases. Patients with other posterior eye segment diseases (macular degeneration, the central retinal artery, vein, or branches occlusion) or anterior and posterior eye segment diseases that did not allow fundus visualization and photography (previous ocular trauma, acute infection, ocular surface diseases and irregularities, mature cataract, opacities, and vitreous hemorrhages) and patients with poor cooperation were not included in the study.

### 2.3. Retinological Examinations, Medication, and Follow-Up

After the best-corrected visual acuity (BCVA) testing and pupil dilation with eye drops containing 0.5% tropicamide, each patient underwent color-fundus photography of two fields (macular field, disk/nasal field) of both eyes with a standard 45° fundus camera VISUCAM Zeiss according to the EURODIAB retinal photography methodology [18]. After that, the first, M.T., and the second author, T. Babić, two retina specialists, independently graded the photographs and assigned a DR grade using the Proposed international clinical diabetic retinopathy and diabetic macular edema disease severity ratings [19]. Since there was no case where the experts assigned different grades, there was no need for the third grader. In patients with clinical indications, optical coherence tomography (OCT) of the macula was performed on the same day, and fluorescein angiography (FA) was performed within a week. OCT of the macula was performed using Spectral Domain OCT (SOCT Copernicus REVO, Optopol technology, Zawiercie, Poland), and FA was obtained with the standard 45° fundus camera VISUCAM Zeiss (Carl Zeiss Meditec AG, Goeschwitzer Str. 51–52, Jena, Germany).

Patients with microaneurysms, hemorrhages, and hard exudates were advised to apply citicoline (Omk1^®^) eye drops three times a day continuously and scheduled for their next follow-up visit within three to four months, followed by a visit six months later.

During the complete study period, the third and fourth authors, T.B. and S.Lj., diabetologists, were consulted regarding the metabolic control and renal function of each patient.

## 3. Results

Although all included patients with nonproliferative DR (NPDR) and hard exudates in the macula showed a reduction or complete disappearance of hard exudates with the application of citicoline (Omk1^®^) eye drops, due to the limitation of the manuscript size, we present here only four patients.

### 3.1. Mild NPDR with Hard Exudates in Type 1 Diabetes

**Figure 1:**
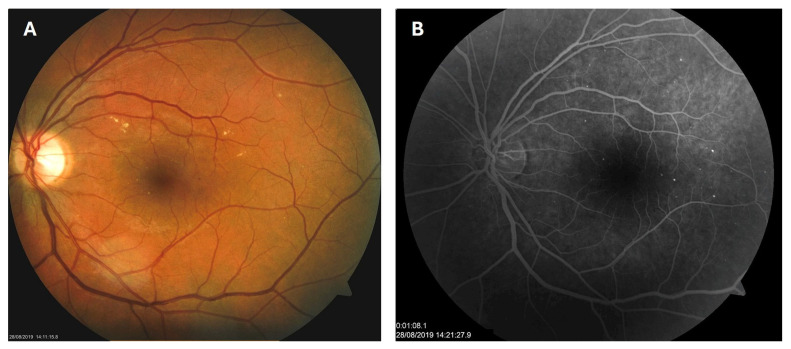
Color photo (**A**) and fluorescein angiogram (**B**) of the left macula of patient 1 in August 2019.

**Figure 2:**
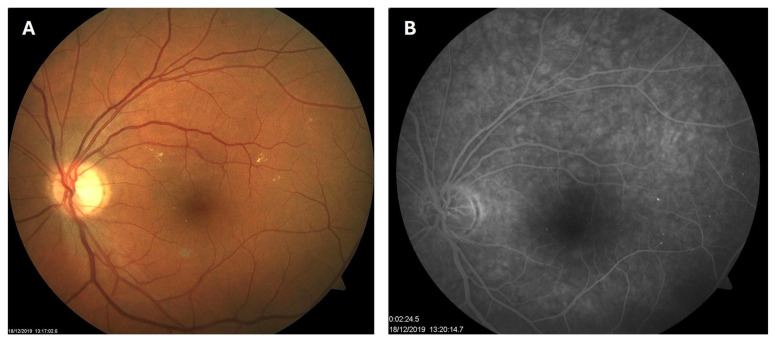
Color photo (**A**) and fluorescein angiogram (**B**) of the left macula of patient 1 in December 2019.

**Figure 3:**
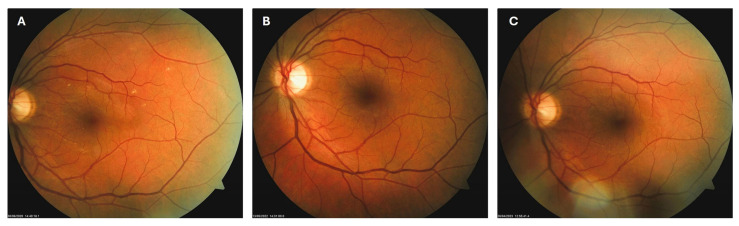
Color photo of the left macula of patient 1 in June 2020 (**A**), May 2022 (**B**) and April 2023 (**C**).

Patient 1: A 42-year-old male with a 28-year history of type 1 diabetes mellitus (T1DM) was diagnosed with mild NPDR during a routine fundus examination in August 2019. His best-corrected visual acuity (BCVA) in both eyes was 1.0, though hard exudates and microaneurysms were observed in his left macula (Figure 1A,B). At the same time, his laboratory results indicated good glycemic control (HbA_1_c 6.0%), but elevated lipid profile levels (total cholesterol 7.75 mmol/L, LDL 5.72 mmol/L, HDL 1.23 mmol/L, and triglycerides 1.73 mmol/L). His blood pressure was 135/85 mmHg, and the albumin-to-creatinine ratio (A/C ratio) was 1.6 mg/mmol. Due to the signs of DR in his left macula, he was advised to use citicoline eye drops three times a day for a period of three months. In December 2019, although the number and the size of hard exudates did not change significantly, there was a decrease in the number of microaneurysms on fluorescein angiography (Figure 2A,B). This was the time of the coronavirus pandemic, so his fundus examinations were rare. However, he continued to use the citicoline drops according to the guidance, three times a day in the left eye. In June 2020, the hard exudates in his left macula had diminished (Figure 3A), and in May 2022, they had completely disappeared (Figure 3B). On his last visit in April 2023, he had no signs of DR and no visible hard exudates in his left macula (Figure 3C). At that time, his laboratory results remained unchanged. His glycemic control was good (HbA_1_c 6.5%), while the lipid profile was still elevated (total cholesterol 7.4 mmol/L, LDL 5.3 mmol/L, HDL 1.2 mmol/L, and triglycerides 1.9 mmol/L). His blood pressure was 130/80 mmHg, and the A/C ratio slightly increased to 2.0 mg/mmol.

### 3.2. Moderate NPDR with Hard Exudates in Type 2 Diabetes

**Figure 4:**
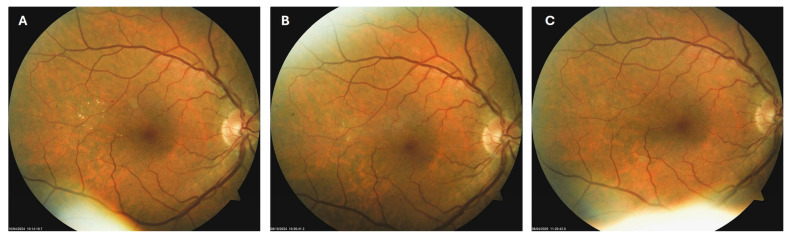
Color photo of the right macula of patient 2 in October 2023 (**A**), February 2024 (**B**) and March 2025 (**C**).

Patient 2: A 54-year-old male with a 20-year history of type 2 diabetes mellitus (T2DM) was diagnosed with moderate NPDR during a routine fundus examination in October 2023. In the right macula, he had microaneurysms, retinal hemorrhages, and hard exudates (Figure 4A). He was immediately advised to apply citicoline eye drops three times a day into his right eye. During the follow-up visit in February 2024, the number and size of hard exudates in his right macula had decreased significantly (Figure 4B), while at the last visit in March 2025, they had disappeared entirely (Figure 4C). However, macular hemorrhages remained unchanged despite the treatment.

**Figure 5:**
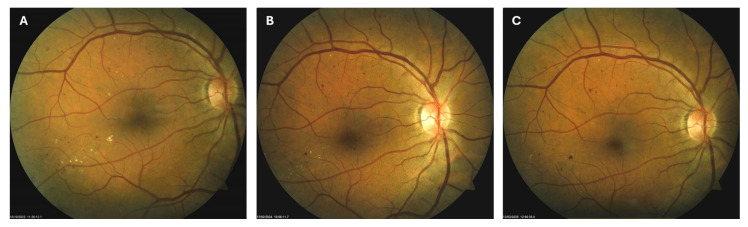
Color photo of the right macula of patient 3 in April 2024 (**A**), October 2024 (**B**) and March 2025 (**C**).

Patient 3: A 66-year-old male with T2DM for 25 years was diagnosed with moderate NPDR in April 2024. Similarly to the previous case, on the right macula, he had microaneurysms, retinal hemorrhages, and hard exudates (Figure 5A), for which he was also advised to apply citicoline eye drops three times a day into the right eye. At the next visit in October 2024, the number and size of hard exudates in his right macula had diminished significantly (Figure 5B), while at the last visit in March 2025, they had disappeared entirely (Figure 5C).

Both patients had good control of glycemia, lipid profile, and blood pressure at baseline and during follow-up, and did not have substantial renal impairment.

### 3.3. Severe NPDR with Center-Involved Diabetic Macular Edema and Hard Exudates in Type 1 Diabetes

**Figure 6:**
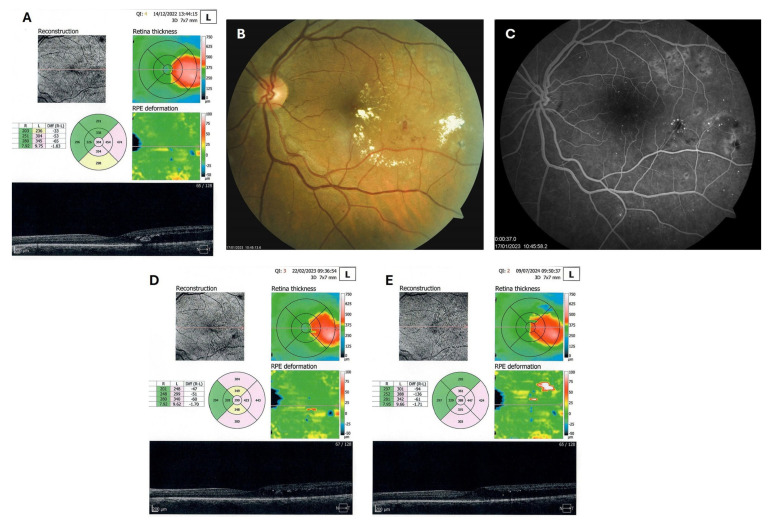
OCT (**A**), color photo (**B**), and fluorescein angiogram (**C**) of the left macula of patient 4 in December 2022/January 2023, and OCT of the left macula on follow-ups in February 2023 (**D**) and July 2024 (**E**).

**Figure 7:**
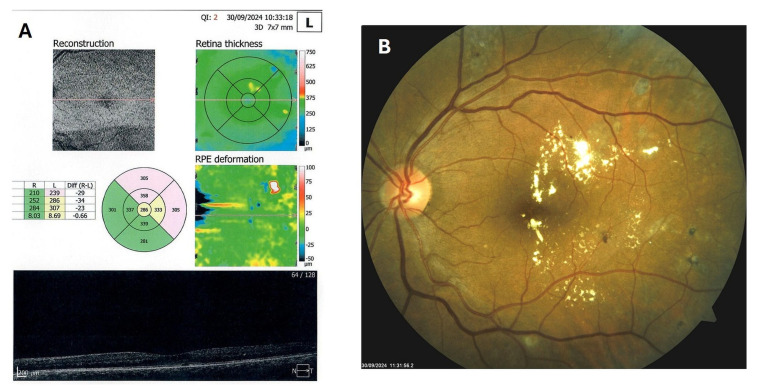
OCT (**A**) and color photo (**B**) of the left macula of patient 4 in September 2024.

**Figure 8:**
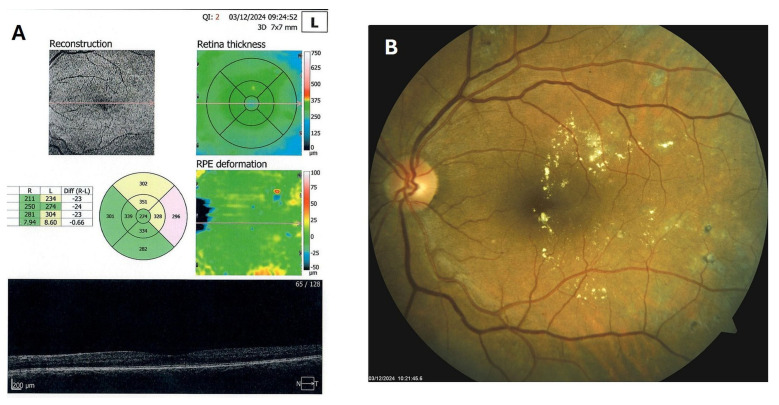
OCT (**A**) and color photo (**B**) of the left macula of patient 4 in December 2024.

**Figure 9:**
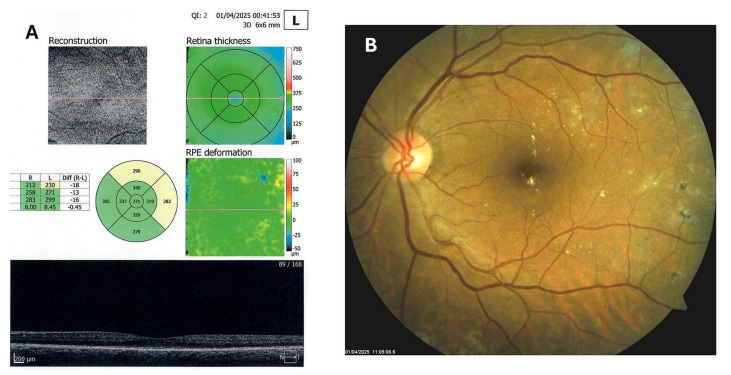
OCT (**A**) and color photo (**B**) of the left macula of patient 4 in April 2025.

Patient 4: A 53-year-old male with a 40-year history of T1DM was diagnosed with severe NPDR and center-involved diabetic macular edema (CI-DME) in December 2022 (Figure 6A–C). At that time, his BCVA in the left eye was 0.8. Throughout 2023 and 2024, he underwent multiple treatments, including six intravitreal injections (IVT) of bevacizumab and six IVT of aflibercept, along with retinal laser photocoagulation. However, the last IVT of aflibercept was in June 2024, and the final laser treatment was in July of the same year. Despite these interventions, there were no significant improvements (Figure 6D,E), and the patient opted to discontinue both IVT and laser treatments. Considering the condition of his left macula and decision about treatment, he was recommended to use citicoline eye drops, three times a day, continuously. On the patient’s next visit in September 2024, NPDR and CI-DME were still present, with a BCVA of 0.8. However, since the hard exudates in his left macula had reduced (Figure 7A,B and Figure 8A,B), he was advised to continue with citicoline treatment. In April 2025, a follow-up examination revealed an improvement in his BCVA, which had increased to 1.0, and in his macula OCT findings, with the hard exudates almost completely disappearing (Figure 9A,B). Assuming all this, there was no need for IVT treatment, but he was recommended to continue the therapy with citicoline eye drops in the same regimen.

In this patient, similarly to the previous patients, the metabolic control of diabetes, blood pressure, and A/C ratio remained stable from baseline to the end of the follow-up period (14 December 2022. HbA_1_c 7.2%, total cholesterol 4.0 mmol/L, LDL 2.4 mmol/L, HDL 0.9 mmol/L, and triglycerides 1.6 mmol/L, BP 130/85 mmHg, and A/C ratio 1.1 mg/mmol; 30 September 2024. HbA_1_c 7.2%, total cholesterol 4.3 mmol/L, LDL 2.7 mmol/L, HDL 1.0 mmol/L, and triglycerides 1.4 mmol/L, BP 135/80 mmHg, and A/C ratio 2.0 mg/mmol; 1 April 2025. HbA_1_c 7.0%, total cholesterol 3.9 mmol/L, LDL 2.2 mmol/L, HDL 0.9 mmol/L, and triglycerides 1.2 mmol/L, BP 130/80 mmHg, and A/C ratio 1.8 mg/mmol).

## 4. Discussion

In patients with NPDR macular hard exudates, the application of citicoline (Omk1^®^) eye drops three times a day for at least six months to a year resulted in a reduction or complete disappearance of HE. These results highlight the patients’ response to citicoline therapy over time, suggesting a promising avenue for future therapeutic interventions and offering a more comprehensive approach to managing DR and reducing its burden. Several mechanisms may explain the association between glutamate dysregulation and the development of HE in DR [20,21]. First, excessive glutamate levels, via oxidative stress and inflammation, disrupt the BRB and increase permeability, allowing the leakage of plasma components, including lipoproteins, into the retinal layers, which, over time, aggregate and form the HE. Second, glutamate excitotoxicity upregulates the expression of VEGF, which exacerbates capillary damage and promotes the leakage of lipids and proteins, contributing to the formation of HE. Third, glutamate dysregulation triggers inflammatory pathways, leading to the activation of microglia and the release of pro-inflammatory cytokines, which contribute to ECM remodeling and chronic inflammation, creating an environment conducive to the deposition and persistence of HE.

Understanding the link between glutamate and hard exudates offers promising opportunities for targeted therapeutic interventions in DR. Today, widely used anti-VEGF therapies not only address VEGF-induced neovascularization but also mitigate vascular leakage and glutamate-related damage, addressing multiple aspects of hard exudate development [22,23]. Furthermore, anti-inflammatory approaches that target microglial activation and pro-inflammatory cytokines may reduce ECM remodeling and improve retinal vascular health, thereby indirectly reducing hard exudates [24,25]. However, intravitreal anti-VEGF or corticosteroid treatment is not indicated in cases of mild or moderate NPDR without CI-DME [26,27]. Thus, for these cases, a more recent approach might involve pharmacological agents that regulate glutamate levels or block its excitotoxic effects, thereby minimizing vascular damage and hard exudate formation.

Citicoline, also known as cytidine-5′-diphosphocholine, is a precursor of both phosphatidylcholine, which is essential for the synthesis of structural phospholipids in cell membranes, and acetylcholine, an important neurotransmitter for cell metabolism [28,29]. In addition to increasing the levels of various neurotransmitters in the central nervous system, citicoline also exhibits a neuroprotective effect, particularly beneficial in neurodegenerative diseases such as Parkinson’s and Alzheimer’s diseases [30,31]. Many experimental studies have investigated the administration of citicoline in various conditions and pathologies [28]. In the field of ophthalmology, improvements in visual function have been noted in patients with glaucoma, DR, non-arteritic ischemic optic neuropathy (NAION), and amblyopia [32,33,34,35]. By inhibiting the phospholipase A2 (PLA2) enzyme, which is responsible for breaking down membrane phospholipids into arachidonic acid, citicoline reduces inflammation, the formation of reactive oxygen species (ROS), and neuronal damage [36]. It has a cytoprotective effect against glutamate and high glucose neurotoxicity, as observed in primary cultured retinal cells [37]. Additionally, citicoline has a neuroprotective effect against neurotoxic damage induced by kainic acid (KA), a glutamate receptor agonist, that causes excitotoxic damage in retinal cells [38] and in vitro models of retinal neurodegeneration [39]. Bogdanov et al. suggested that the topical use of citicoline in a liposomal formulation may prevent glial activation and apoptosis, making it a promising new strategy for treating the early stages of DR [40]. While more research is needed, early clinical studies suggest that citicoline eye drops may stabilize or improve visual function and reduce neuroretinal degeneration in patients with mild DR. Parisi et al. [41] reported the results of a pilot study evaluating the long-term efficacy of citicoline and vitamin B12 eye drops on macular function in T1DM with mild signs of NPDR. Patients treated with citicoline and vitamin B12 eye drops for 36 months showed an improvement of macular bioelectrical responses, while those receiving a placebo experienced a decline in macular function over the same follow-up period.

Citicoline might complement anti-VEGF treatment by addressing the neurodegenerative aspects of the disease that are not targeted by anti-VEGF, which addresses the vascular component of DR. However, there is still limited data directly investigating the synergistic effect of citicoline with existing treatments in DR and other retinal diseases. Nashine et al., in their study examining the role of citicoline in an in vitro model of age-related macular degeneration (AMD), found that citicoline decreases ROS production and downregulates VEGF gene expression in AMD retinal pigment epithelium cybrid cells [42]. Another study showed that the combination of citicoline, homotaurine, and vitamin D3 had anti-inflammatory and neuroprotective effects in experimental models of DR [43]. Surely, more research is needed to fully understand the synergistic effects of citicoline and anti-VEGF drugs in the treatment of DR, particularly in larger clinical trials and with longer follow-up periods. Regarding our patient 4, and why HE improved after anti-VEGF failure, we do not have an exact pathogenetic, scientific, and evidence-based answer and explanation. Anti-VEGF medications, such as bevacizumab, ranibizumab, and aflibercept, bind to and neutralize VEGF, preventing it from interacting with its receptors on endothelial cells. They reduce neovascularization and leakage from blood vessels, thereby primarily leading to a resolution of retinal edema, intraretinal (IR) fluid, and a decrease in central macular thickness (CMT) as observed by OCT [44,45,46]. They were not primarily focused on the resolution of HD. In our patient 4, it might be presumed that the anti-VEGF treatment, which did not yield the expected results for HE and edema, initially still reduced the VEGF and the vascular component of DR. This initial reduction may have created conditions that allowed citicoline, with its neuroprotective effects, to act more effectively through glutamate blockade in the previously prepared area. There is especially the reasonable speculation that “anti-VEGF may create conditions for the action of citicoline”.

Several limitations of this study should be addressed. First, the main limitations of this study are its observational design and small sample size, which limit the generalizability of the results to larger populations and necessitate careful consideration of the findings in broader discussions. However, in our Department of Diabetic Eye Complications, we have over 100 patients with macular HE who were successfully treated with citicoline eye drops and followed up similarly. Still, we were unable to include all of them in the present study. Second, the assessment of HE relied on qualitative analysis of color fundus photography and lacked quantitative measurements using OCT. These limitations need to be overcome in future prospective studies with more rigorous designs and larger sample sizes. Both OCT and fundus photography are used to detect and analyze macular HE, but they offer different perspectives. OCT provides detailed images of the retina, allowing for the identification of hyperreflective foci that may indicate early exudates, while fundus photography offers a broader, two-dimensional view of the retina, highlighting the location and extent of visible exudates [47,48,49]. Initially, when we began prescribing citicoline eye drops, we did not expect their positive impact on the HE, and earlier published studies had shown only improvements in macular bioelectrical responses and morpho-functional retinal changes in patients with mild signs of NPDR [41,50], so we did not quantitatively monitor the size of HE. Considering the established positive effect of citicoline on HE, we will soon design and initiate a new research study in which we will quantitatively monitor the resolution of hard exudates under the influence of citicoline.

## 5. Conclusions

These cases present the complexities of managing diabetic retinal diseases and highlight the patients’ response to citicoline therapy over time. Although this preliminary study reports for the first time a potential positive effect of citicoline on hard exudates in DR, given the limitations mentioned above, this conclusion requires further verification in larger studies. Citicoline is known to reduce the release of glutamate and its toxicity to nerve cells. Furthermore, it increases the secretion of glutathione, reduces inflammation, and stimulates nerve cell growth. Citicoline (Omk1^®^) eye drops are indicated in glaucomatous patients as an adjunct to hypotensive therapy. While the direct impact of citicoline on the resolution of macular hard exudates remains unclear, its potential to influence key pathways, such as glutamate toxicity, oxidative stress, and inflammation, suggests a promising avenue for future therapeutic interventions. Incorporating citicoline into treatment paradigms could complement existing anti-VEGF, antioxidant, and anti-inflammatory therapies, offering a more comprehensive approach to managing DR and reducing its burden. In our study, all patients had good control of glycemia, lipid profile, and blood pressure at baseline and during follow-up, and did not have substantial renal impairment. However, considering the small number of patients, we plan to conduct a new prospective study to perform quantitative analysis and statistical tests on the improvement rate and time distribution of hard exudates, thereby further confirming the results of this study.

## Data Availability

The data presented in this study are available at a specific request from the corresponding author.
